# Fabrication of Micro-Parts with High-Aspect Ratio Micro-Hole Array by Micro-Powder Injection Molding

**DOI:** 10.3390/ma11101864

**Published:** 2018-10-01

**Authors:** Changrui Wang, Zhen Lu, Kaifeng Zhang

**Affiliations:** 1 The 14th Research Institute of China Electronics Technology Group Corporation, Nanjing 210039, China; 2 School of Materials Science and Engineering, Harbin Institute of Technology, Harbin 150001, China; luzhenhit@hit.edu.cn (Z.L.); kfzhang@hit.edu.cn (K.Z.)

**Keywords:** micro-powder injection molding, high-aspect ratio micro-hole array, microstructure, sintering, shrinkage

## Abstract

The present study investigated high-aspect ratio micro-hole array parts which were made by ZrO_2_ micro-powder with different particle sizes and micro-powder injection molding technology. It analysed the influence of particle sizes on feedstock, debinding and sintering of ceramic nozzles with multi-micro-holes. The forming quality of ceramic nozzles with multi-micro-holes was discussed in this paper. The results show that the two mixed ZrO_2_ feedstocks have fine uniformity. The average deviation of the feedstock made with 200 nm powder was −2%, and the average deviation of the feedstock made with 100 nm powder was −7.1%. The sample showed certain sintering characteristics which provided better strength (11.10 MPa) to parts after debinding. The linear shrinkage and the density of the two powder samples at different sintering temperatures increased as the sintering temperature increased. If the temperature continued to increase, the linear shrinkage and the density decreased. The highest hardness and flexural strength values of the ZrO_2_ sample with 200 nm powder used were: 1265.5 HV and 453.4 MPa, and the crystalline particle size was 0.36 μm. The highest hardness and flexural strength values of the ZrO_2_ sample with 100 nm powder used were: 1425.8 HV and 503.6 MPa, and the crystalline particle size was 0.18 μm. The ceramic nozzles with multi-micro holes shrunk to nearly the same axial, radial and circumferential directions during sintering. After sintering, the roundness of ceramic micro-hole met the user requirements, and the circular hole had a high parallelism in the axial direction. The micropore diameter was 450 ± 5 μm, and it was possible to control the dimensional accuracy within 1.5% after sintering. The study presented a superior application prospect for high-aspect ratio micro hole array parts in aerospace, electronics and biomedicine.

## 1. Introduction

With the rapid development of product miniaturization, the demand for micro-bearings, micro-cams, micro-holes, micro-gears, microscope heads, micro-tubes, micro-motors and other components is increasing [1,2,3]. As a typical widely used microstructure, micro-holes act as transfer and exchange media in the form of micro-channels or nozzles that can be used as fuel injection nozzles, spinnerets, jet turbine blade cooling holes, printer jets, fiber optic connections, micro-structures of an array hole in a micro-pump, and micro-hole structures in fiber optic connectors and biomedical filters [4,5,6,7]. In the aerospace, automation, and bioscience fields, fuel nozzles and micro-holes are supposed to have the qualities of wear resistance, high temperature resistance, and high pressure resistance. Compared with metal micro-holes, ZrO_2_ ceramic micro-holes have the advantages of high hardness, good wear resistance, high temperature resistance and acid and alkali corrosion resistance, and thus, have superior application prospects in aerospace, electronics, and biomedicine [8]. However, ceramic materials have high brittleness, poor formability, and are difficult to process, resulting in high costs, poor precision, and poor surface quality [9,10]. As a near-net-shape technology, micro-powder injection molding has the characteristics of low cost, high precision and high efficiency. It can produce parts with complex shapes on a large scale and is used to make ceramic micro-holes, in particular, it has great advantages in the production of array ceramic micro-holes [11,12,13,14].

The right powder should be selected before the experiment. Poh L. used thermogravimetric analysis (TGA) and differential scanning calorimetry (DSC) to analyse powder distribution within the ceramic injection molding green body [15]. The reduction in the particle size of powder decreased the powder loading due to the bimodal packing effect [16]. Larger particles with a wider distribution in size showed relatively lower levels of viscosity and improved the flow stability of the feedstock. So, the powder should have wider distribution in accordance with the requirements of the micro-powder injection molding process [17,18,19]. Next, suitable molds should be designed and manufactured. Wang Q. studied the effects of the mold dimensions on the rheological properties of the feedstock and the inner wall of the mold was shown to be the key factor for injection molding [20]. A separation mold system was studied in order to prepare the micro-sized piezoelectric structure [21]. Micro-powder injection molding includes main four steps: feedstock preparation, molding, debinding and sintering [22,23]. The uniformity of the feedstock has a large effect on the quality of the injection and sintered parts. If the feedstock is not uniform after mixing, the result will be a distortion of the sintered parts. Previous work in our laboratory acquired consistent feedstock which met the requirements for micro-powder injection molding by using a paraffin base binder [24,25]. Injection molding is a key step in the formation of high-quality parts. It has been found that the plasticizing temperature, injection pressure, mold temperature and holding time have a great influence on the quality of injection molding parts [26]. In our preliminary research, appropriate injection molding parameters were also discussed, and a suitable process was determined [27]. The debinding process is also one of the key steps in micro-powder injection molding. The main purpose is to remove the binder in the billet by micro-powder injection molding. There are several methods by which the debinding process can remove the binders, such as solvents debinding, thermal debinding and catalytic debinding [28,29,30]. The most commonly used is the thermal debinding process, which makes the binder evaporate by heating. Ani S.M. studied debinding binders in two stages using ceramic injection molding parts which can prevent defects from forming [31]. Liu et al. analyzed the influencing factors of the paraffin-based binder used in zirconia injection molding in the debinding process, pointing out that the debinding process is mainly affected by the loading amount, the thickness of the sample and the rate at which the volatile binder diffuses to the surface [32]. Preliminary experimental research found that thermal debinding in an ambient atmosphere feasible for micro-powder injection molded parts [33]. Sintering is the last main step of micro-powder injection molding. By heating, the voids in the parts are reduced, and the size of the parts shrink and gain sufficient strength. In addition, the parts are densified to obtain higher density. Foudzi et al. investigated the micro-powder injection molding of yttria-stabilized zirconi [34]. Ni et al. analyzed the influences of the sintering temperature and sintering time on the mechanical properties and structure of micro-powder injection molded parts [35].

What is more, the finite element method has been used to simulate the rheological characteristics of feedstock and filling during the molding, debinding and sintering processes of micro-powder injection molding. This can be used to estimate the injection molding parameters, relative density, shrinkage, and relative density. It can provide information that is difficult to measure, such as instantaneous temperature, pressure, viscosity and velocity which can guide the study of powder injection molding better by combining simulation with experiment [36]. Heaney et al. simulated the shrinkage of debound and sintered micro-powder injection molded parts by the finite element method. Through a comparison with experimental results, it was found that the reliability of the simulation was very high, which is a good guiding force and is significant for follow-up design and research [37]. Somasundram et al. presented a new mathematical model to simulate the isothermal debinding of micro-powder injection molded cylindrical parts. The simulation results are in good agreement with the experimental results, and a simplified model was proposed to better guide the design [38]. Wang et al. built a cellular automata model of composite ceramic material which contained sintering additives and pores based on the grain boundary energy theory and the grain growth boundary curvature driving force theory. This model can be used to simulate sintering densification processes for ceramic materials which contain sintering additives, especially for the sintering densification process involving powder injection molding [39].

Yu et al. carried out a micro-injection molding test of zirconia micro-gears to obtain a zirconia micro-gear with good performance [40,41]. Piotter et al. [42] and Gietzelt et al. [43] successfully produced high-precision ZrO_2_ screws and micro-gear pumps with a three-dimensional thread structure, which opened up a wider application space for micro-powder injection molding ZrO_2_ parts. However, there have been few studies on ZrO_2_ ceramic micro hole parts with a high-aspect ratio.

Based on the micro-powder injection molding of a high-aspect ratio ceramic micro-hole nozzle, this paper systematically analyzed the influences of different particle sizes on the micro-powder injection molding process, debinding process and sintering process of a ceramic micro-hole. It is designed suitably for ceramic micro-hole molds, explores the rheological properties of the feedstock melts, and studies the laws of the debinding, sintering and mechanical properties.

## 2. Experimental Methods

**Figure 1:**
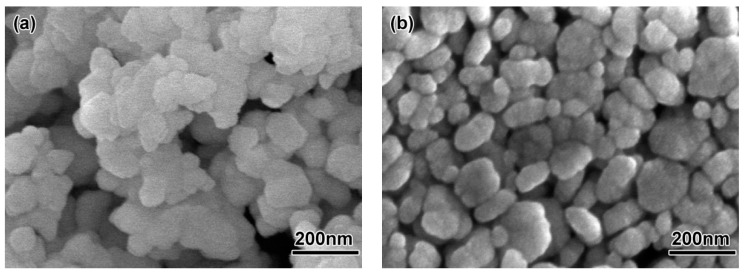
Scanning electron microscope (SEM) micrographs of powders: (**a**) 200 nm, (**b**) 100 nm.

**Figure 2:**
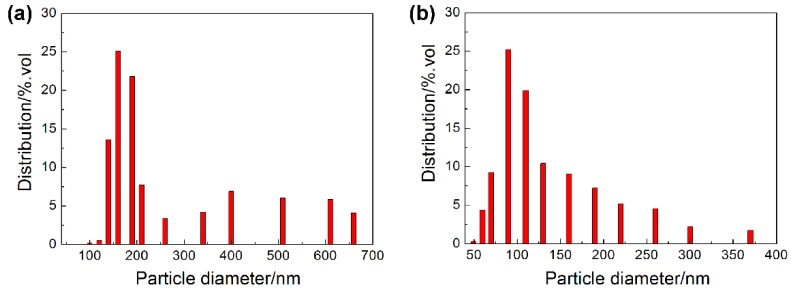
Size distribution of powders: (**a**) 200 nm, (**b**) 100 nm.

In order to study the effect of powder size on feedstock, injection molding, debinding and sintering, two powder sizes were used in this experiment. The injection molding of 3 mol Y_2_O_3_ partially stabilized tetragonal phase ZrO_2_ micro-powder with a mean particle size of 200 nm produced by Hebei Pengda New Material Technology Co., Ltd. (Handan, China) and the micro-powder with a mean particle size of 100 nm, was produced by Dongguan Nanbo Ceramic Technology Co., Ltd. (Dongguan, China). As shown in Figure 1, the powder particles were spherical, and the powder had a certain level of agglomeration but it was not obvious. The composition of the ZrO_2_ ceramic powder for the experiment was shown in Table 1. The powder was characterized by a HORIBA laser scattering particle size distribution analyzer (LA-920, Kyoto, Japan), as shown in Figure 2. The size distribution of the powder had a relatively wide range in accordance with the requirements of the micro-powder injection molding process.

For the ceramic nozzles with multi-micro-holes, the binder ratio (mass ratio) was Paraffin wax (PW): Polypropylene (PP): Stearic acid (SA) = 75:20:5. The feedstock was mixed on a 2 L double planetary mixer (HY-DLH2L) manufactured by Guangzhou Hongyun Machinery Factory (Guangzhou, China). Then, extrusion granulation was carried out with a single screw extruder. The specific process was as follows: firstly, the pre-weighed ceramic powder was added. It was heated to 175 °C and kept warm for 30 min. Then the double planetary rotor, rotating at a speed of 20 r/min was started so that the whole powder was heated uniformly. Then, it was rotated at 40 r/min and PP was added to the barrel gradually and mixed for 30 min. Then PW and SA were added and mixing continued for 30 min. Then, the mixture was cooled and the mixed feedstock was removed and extruded several times on the extruder to produce a cylinder feedstock with a length of 2–3 mm.

**Figure 3:**
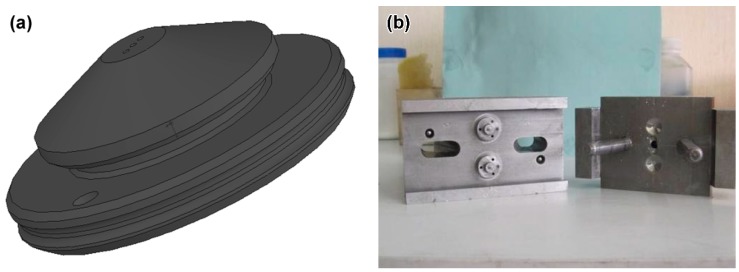
Schematic diagram (**a**) and micro mold (**b**) of ceramic nozzles with multi-micro holes.

When the feedstock granule was being produced, the temperatures at the feedstock inlet, the intermediate heating zone, and the product outlet were set to 175 °C, 170 °C, and 165 °C, respectively. Injection molding was done with a Babyplast 6/10 injection molding machine made in Barcelona, Spain. The schematic diagram of the ceramic micro-hole nozzle and the mold structure for the injection molding were shown in Figure 3. The mold core had three micro-cylinders with an aspect ratio of 10 or more. The micro-holes on the ceramic micro hole nozzle were required to have a desired diameter of 450 μm after sintering. As required by the experimental powder loading, the micro cylinder diameter on the mold core was scaled up. This experiment used a set of n injection molding methods involving heating and cooling. In order to meet the requirements of high mold temperature, which means that the heating rate must be increased during micro-injection, electric heating rods and multi-channel water circulation cooling were used in this process.

The injection molding blank was degreased in an air furnace, and the debinding process was as follows: heating to 180 °C at 0.25 °C/min; this was maintained for 2 h, at which time the low melting point PW melted and decomposed, and migrated to the surface of the blank to volatilize. Heating continued 380 °C at the same heating rate and this was maintained for 2 h. At this time, a through gap formed, and then heating to 500 °C was carried out at the same heating rate and maintained for 2 h, at which time the remaining binder was completely removed. Finally, it was heated to 900 °C at 2 °C/min and kept for 2 h. This stage was mainly a pre-sintering process to ensure that the degreased parts had durable strength. Finally, the densified ceramic parts were obtained by sintering in a high temperature furnace, and the densities of the sintered samples were measured with the Archimedes drainage method. For the measurement of mechanical properties, a strip shape of 4 mm × 4 mm × 20 mm and a pellet sample of 25 mm in diameter and 20 mm in thickness were made; bending strength and fracture toughness tests were performed on an Instron-1186 universal testing machine (Instron, Cincinnati, OH, USA). The hardness test was carried out using an HVS-5 micro- hardness tester (Huayin, Laizhou, China), measuring a load of 5 kg and holding the pressure for 10 s. The above data were measured in 6 groups to get an average figure. The surface roughness of the sample was measured by a LEXTOLS 3000 laser scanning confocal microscope (Olympus, Tokyo, Japan), and the structure was observed with S-4700 scanning electron microscope (Hitachi, Tokyo, Japan).

## 3. Results and Discussion

### 3.1. Feedstock Characteristics

The larger the particle size is, the larger the critical powder loading will be. For spherical particles of a single size, the maximum stacking density can reach more than 70%. However, as the particle size decreases, the particle size of the powder decreases by 50%, and the specific surface increases one-fold, as shown in Formula (1):

(1) $$\frac{S_{b}}{S_{s}}=\frac{D_{s}}{D_{b}}\;$$

In the formula, assuming the particles are spherical, *S_b_*, *D_b_*, *S_s_* and *D_s_* are the specific surface area, diameter of large particles, the specific surface area and diameter of the small particles, respectively. The critical powder loading is about 60 vol% for ceramic powders with a particle size of less than 1 m [44]. When the critical powder load is used to mix the feedstock, the viscosity of the feedstock is high due to the high powder content. It is difficult to fill and easily causes the separation of the powder and binder; in addition, it is also difficult to demould. It is easy to fracture the specimen and cause damage to the formed surface. For this reason, the actual powder loading is lower than the critical powder loading. After many experiments, the optimum powder contents of various powders used in the experiment were 55 vol% using a 200 nm powder, and 50 vol% using a 100 nm powder.

**Figure 4:**
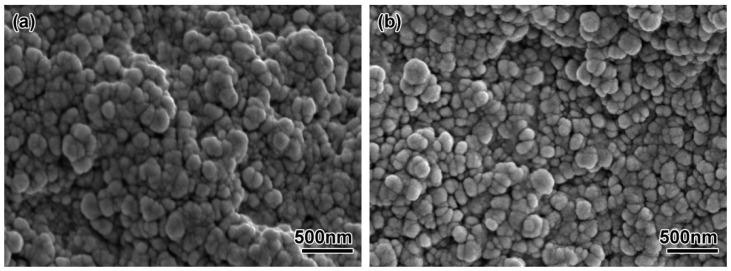
SEM micrograph of the ZrO_2_ feedstock with different particle sizes: (**a**) 200 nm, (**b**) 100 nm.

The uniformity of the feedstock has a large effect on the quality of the formed part. If the feedstock is not uniform after mixing, a density gradient will appear after injection molding, and the void gradient will occur after debinding; these effects are difficult to eliminate in subsequent work, and finally cause the distortion of the sintered parts. Figure 4 shows the fracture morphology of ZrO_2_ feedstock with different powder diameters. It can be seen that the ceramic particles in the two feedstocks were evenly distributed, and the surface was covered with a thin layer of polymer. Compared with the 100 nm powder, the uniformity of feedstock made by 200 nm powder was better, mainly because the particle size of the powder was reduced and the specific surface area increased. Although the powder loading was reduced to 45 vol%, the feedstock viscosity was still large, and the mixture feedstock had micro-holes remaining. The theoretical density of the feedstock made with the 200 nm powder was 3.76 g/cm^3^, and the theoretical density of the feedstock made with the 100 nm powder was 3.50 g/cm^3^. Table 2 showed the actual measured density deviations of different feedstock. Each feedstock was randomly chosen from six samples for measurement. The results showed that the density of each feedstock did not change much, so it could be seen that the two mixings of ZrO_2_ feedstock had better uniformity. The actual measured densities were lower than the theoretical density. The average deviation of the feedstock made with 200 nm powder was −2%, and the average deviation of the feedstock made with 100 nm powder was −7.1%. It was mainly because the air inevitably remained and formed air holes when mixing, which affected the density of the feedstock.

**Figure 5:**
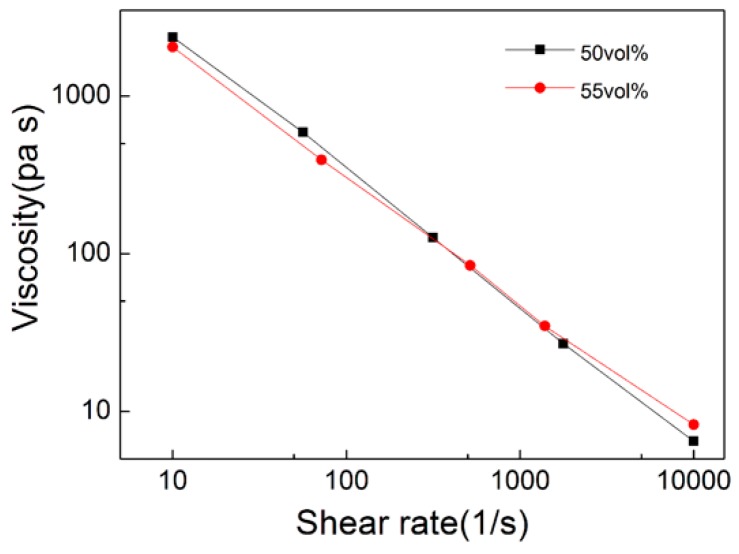
Apparent viscosity of the ZrO_2_ feedstock versus shear rate at 180 °C.

Figure 5 shows the relationship between viscosity and shear rate at different shear strain rates. It can be seen that the viscosity of the feedstock decreased with temperature and shear rate increased. The two groups of feedstock had pseudo-plastic rheological behavior and showed shear thinning which met the requirements of micro-powder injection molding.

### 3.2. Microstructure and Mechanical Properties

Thermal debinding generally consists of three stages, the initial stage, the intermediate stage and the final stage. At the initial stage, when the debinding temperature is heated to a certain temperature, the melting point of a certain binder is reached, and the binder begins to melt. Continuously increasing the temperature will cause the binder to decompose into gas. The gas on the billet surface will go directly into the surrounding atmosphere, thus forming small voids on the billet surface. In the intermediate stage, the surface-to-interior gaps are formed at a distance due to the volatilization of the binder on the billet surface and the internal gas migration to the billet surface. At the final stage, most of the binder has been removed, but the debinding temperature needs to be further increased to ensure that the binder is completely removed.

**Figure 6:**
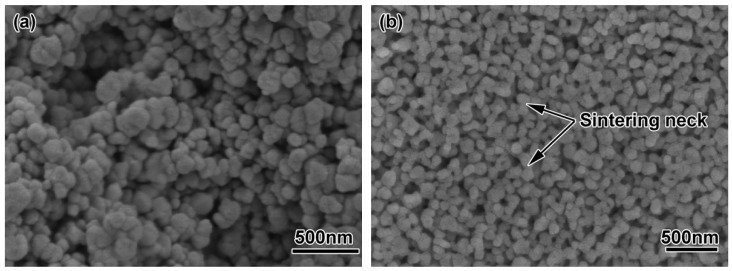
SEM morphology of debound ZrO_2_ samples using different powders: (**a**) 200 nm; (**b**) 100 nm.

The micro-structures of the ZrO_2_ samples with different powder particle sizes after thermal debinding are shown in Figure 6. It can be seen from the figure that the ZrO_2_ sample with a powder particle size of 200 nm had certain sintering characteristics. As shown in Figure 6a, there was a small amount of neck formation, but there were some large pores, mainly due to decomposition and gasification of the binder during debinding; the gas pressure made the powder particles rearrange. After the gas was removed, a large amount of pores remained. The flexural strength and linear shrinkage of the parts after debinding were 6.78 MPa and 1.45%, respectively (see Table 3). Figure 6b shows the microstructure of the sample after debinding using a 100 nm ceramic powder. Compared with the 200 nm powder, the sample with smaller diameter powder particles showed better sintering characteristics. There was a large amount of neck formation, and many adjacent particles formed a dumbbell-shaped neck formation. The remaining pore diameter was also smaller than that of the 200 nm powder, and the overall linear shrinkage (1.78%) of the sample was also larger. It showed that the use of ultra-fine or even nano-particles for micro-powder injection molding reduced the occurrence of debinding defects, resulting in parts having durable strength (11.10 MPa) after debinding. Experimental studies have shown that by using the above two micro powders, samples show certain sintering characteristics after debinding. The bending strength and linear shrinkage of specimens with 100 nm diameter were higher than those with 200 nm diameter. This is mainly because the smaller the particle size is, the higher the surface activation energy is, and the better sintering is at the same temperature. Then, the debounded sample had a certain strength, which ensured transported strength without damage. Therefore, the samples made for each feedstock had sufficient strength for subsequent processing.

**Figure 7:**
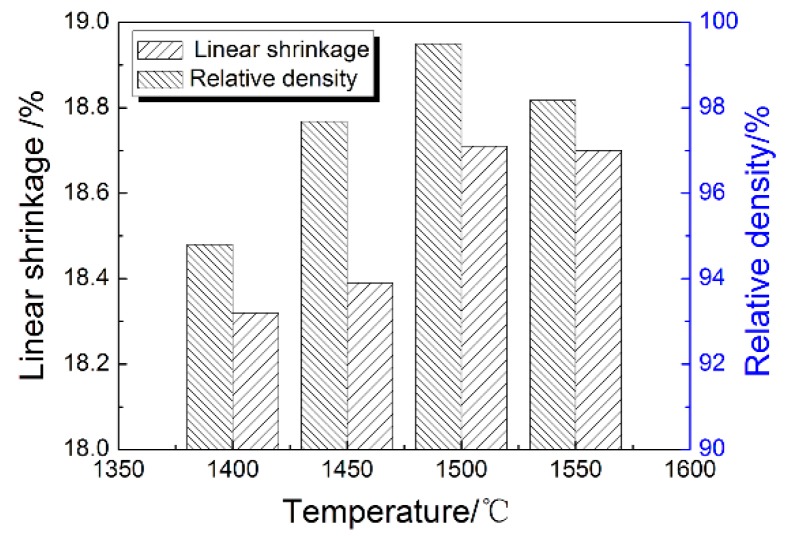
Linear shrinkage and density at different sintering temperatures of ZrO_2_ ceramic nozzles with multi-micro holes using a 200 nm powder.

Figure 7 shows the linear shrinkage and density at different sintering temperatures of ZrO_2_ ceramic nozzles with multi-micro holes using a 200 nm powder. It can be seen that as the sintering temperature increased, both linear shrinkage and density increased. The maximum value occurred at 1500 °C. The temperature continuously increased, and the linear shrinkage and the density decreased. At the beginning, as the sintering temperature increased, the atomic motion became active, providing sufficient activation energy for mass transfer and migration during sintering, which made the pores of the sample shrink and disappear, and the crystalline particle boundaries rapidly migrated and expanded, causing crystalline particle growth. The shrinkage of the sample was increased and the density was increased. The linear shrinkage and density of the samples after sintering at 1400 °C were 17.75% and 94.8%, respectively. When the sintering temperature reached 1500 °C, the linear shrinkage and density reached 18.04% and 99.5%, respectively. However, an excessively high sintering temperature will cause the atomic diffusion coefficient to be too large, and will cause the abnormal growth of crystal crystalline particles. These abnormally grown crystal crystalline particles were several times to several tens-of-times the normal crystalline particle size. When an abnormal growth occurred, the crystal crystalline particles rapidly consumed and fused with the surrounding small crystal crystalline particles, and surrounded many pores, forming internal crystal pores, which were difficult to eliminate in the subsequent stage, thereby influencing the shrinkage and the density of the sample after sintering was reduced. The linear shrinkage and density of the sample after sintering at 1550 °C were 17.96% and 98.18%.

Therefore, linear shrinkage and density increased first and then decreased with the increase of sintering temperature due mainly to the following reasons [41]. At the initial stage of sintering, under the action of sintering driving force, the powder particles rearrange, the sintering neck forms, the large void disappears, and the sintering neck also grows. Neck growth and pore shrinkage reduce the distance between particles, which increases both the sample’s density and shrinkage [45]. With the increase of sintering temperature, the sintering neck continues to increase. The grain boundaries and pores migrate through lattice diffusion and grain boundary diffusion, and the grains begin to grow. The voids in the parts form connected pores on the surfaces of grain boundaries, and a large number of voids begin to disappear. If the sintering temperature is too high or too fast, the diffusion coefficient of atoms will be too large, and abnormal growth of grains will occur. These abnormal growth grains are several to tens-of-times the normal grain size. When abnormal growth occurs, the grain consumes and fuses the surrounding small grains rapidly, and will surround a lot of pores, forming an endocrystalline pore, which is difficult to eliminate in the subsequent stages, resulting in decreased shrinkage and density in the sintered sample.

**Figure 8:**
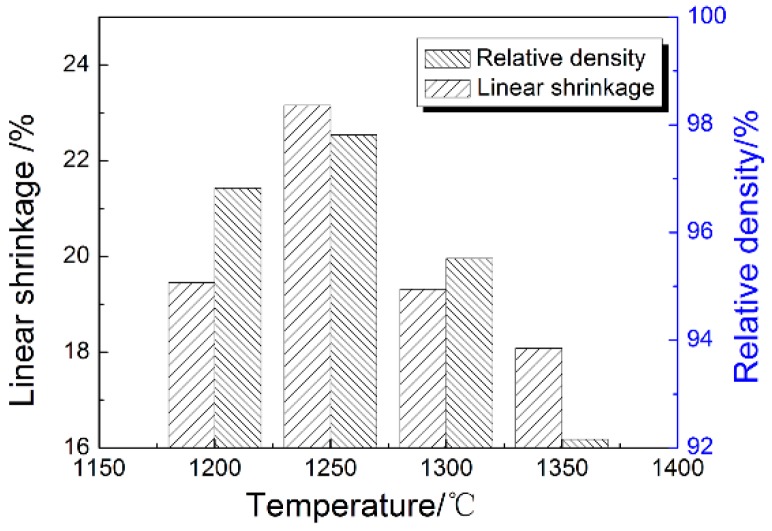
Linear shrinkage and density at different sintering temperatures of ZrO_2_ ceramic nozzles with multi-micro-holes using 100 nm powder.

Figure 8 shows the amounts of linear shrinkage and densities at different sintering temperatures of ZrO_2_ ceramic nozzles with multi-micro holes using a 100 nm powder. With the rise of the sintering temperature, the linear shrinkage and density of the samples increased first and then decreased, reaching maximum values of 20.52% and 98.36% at 1250 °C, respectively. Afterwards, the corresponding shrinkage and density declined greatly with continuous heating, to 20.01% and 93.85% at 1350 °C. The sintering temperature of the nearly-densified sample with 100 nm-powder was much lower than that of the sample with 200 nm powder, up to above 250 °C, since fine powder particles could effectively increase the specific surface area, improve the sintering efficiency and lower the sintering temperature. However, due to the large specific surface area, the powder loading in the preparation of the feedstock decreased, and more voids remained in the degreased sample than in the sample with 200 nm powder. So, therefore, it requires greater shrinkage to achieve higher sintering density. For example, the sample with 100 nm powder was almost fully dense at 1250 °C, when the linear shrinkage of the sample was 20.52%, 2.5% higher than that of the sample with 200 nm powder sintered at 1500 °C. Meanwhile, the high void fraction after debinding more likely caused abnormal grain growth during sintering and thus, led to the deterioration of the properties. Based on the analysis above, it was known that the density and linear shrinkage of the sintered sample showed a consistent relationship. If the powder loading of the feedstock was definite, the linear shrinkage could be estimated accordingly, and the enlargement ratio of the mold cavity could be determined. The optimum densification temperatures of the samples with 200 nm- and 100 nm-powder were 1500 °C and 1250 °C respectively. The variation in the density and linear shrinkage of samples with 100 nm powder were the same as in those with 200 nm powder.

**Figure 9:**
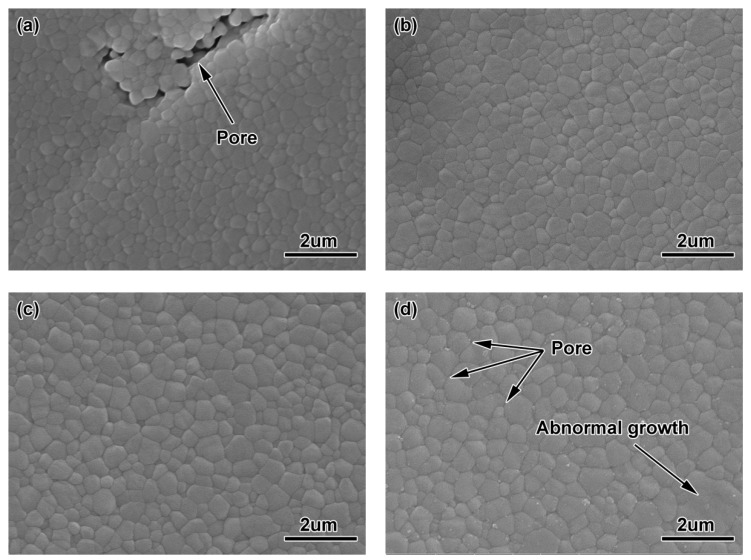
Microstructures of ZrO_2_ samples using 200 nm powders sintered at different temperatures: (**a**) 1400 °C, (**b**) 1450 °C, (**c**) 1500 °C, (**d**) 1550 °C.

**Figure 10:**
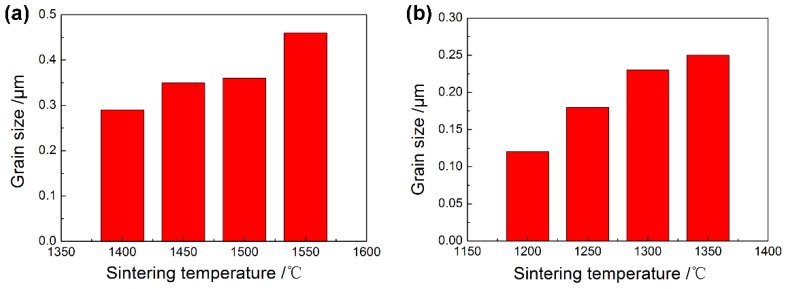
Grain sizes of the ZrO_2_ samples with the milled using different powders sintered at different temperatures: (**a**) 200 nm, (**b**) 100 nm.

Figure 9 shows the microstructure of ZrO_2_ samples with 100 nm powder at different sintering temperatures. In Figure 7a, after sintering at 1400 °C, the grains were shown to be relatively fine, yet still with some larger holes, which makes the sintering density of the sample lower and also affects the mechanical properties of the material. At this time, the average grain size measured by the scratching method was 0.29 μm, as shown in Figure 10. When the sintering temperature rose to 1450 °C (see Figure 9b), the heating intensified the atomic motion, raising the specific surface energy, accelerating the densification process, and increasing the density, eliminating a large number of voids. The grain size ascended to 0.35 μm due to the growth and expansion of some grain boundaries. When the sintering temperature reached 1500 °C (see Figure 9c), the density of the sintered samples increased, the voids were further eliminated, the grains were more uniform, and the average grain size grew slightly to 0.36 μm. The microstructure of ZrO_2_ ceramics obtained at this time were the best. Higher sintering temperatures led to excessive and abnormal grain growth (see Figure 9d), and further resulted in voids at the trigeminal grain boundary and the quadruple grain boundary. The 200 nm-ZrO_2_-powder used in the experiment had a wide particle size distribution. Well-mixed powder in the preparation of feedstock made the density of the samples more uniform; sintering aids were not added. Therefore, the sintering temperature of ZrO_2_ samples using 200 nm powders sintered at 1550 °C was too high, resulting in a fast mass transfer rate at certain grain boundaries so that the holes at the grain boundaries disconnected, and the grain boundaries rapidly expanded to absorb the surrounding grain boundaries.

**Figure 11:**
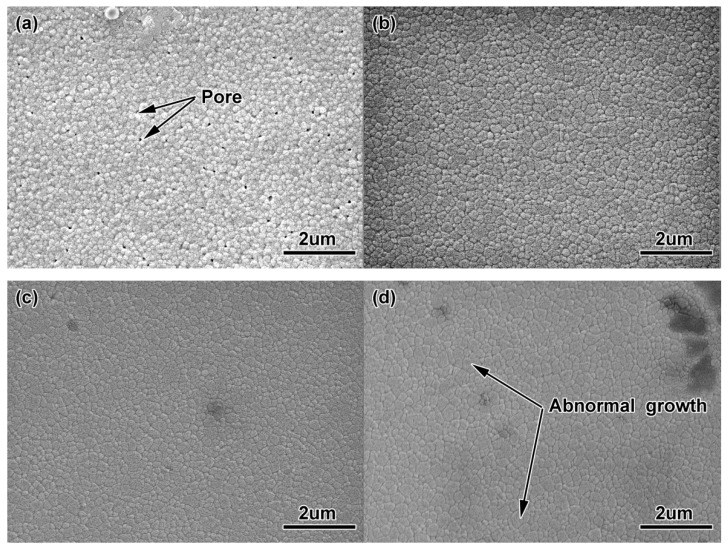
Microstructures of ZrO_2_ samples using 100 nm powders sintered at different temperatures (**a**) 1200 °C, (**b**)1250 °C, (**c**) 1300 °C, (**d**) 1350 °C.

Figure 11 shows the micro-structures of the ZrO_2_ samples with 100 nm powder at different sintering temperatures, which were smaller than those of the ZrO_2_ samples with 200 nm powder. However, at 1200 °C, there were a large number of small voids, and the particle size (0.12 μm) was smaller (see Figure 11a). As the sintering temperature rose, the particle size grew, the density strengthened, and the voids were eliminated. The particle size reached 0.18 μm, as shown in Figure 10b. As the temperature continued to go up, some grains grew abnormally and the voids were surrounded by grain boundaries during sintering, which made them difficult to eliminate and reduced the density, as shown in Figure 11c,d.

**Figure 12:**
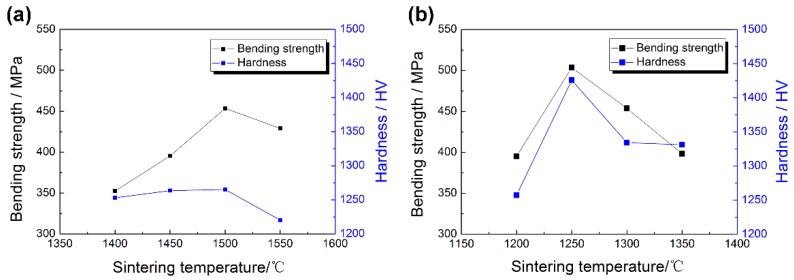
The changes in Vickers hardness of ZrO_2_ ceramic samples with different particle sizes at different sintering temperatures: (**a**) 200 nm; (**b**) 100 nm.

**Figure 13:**
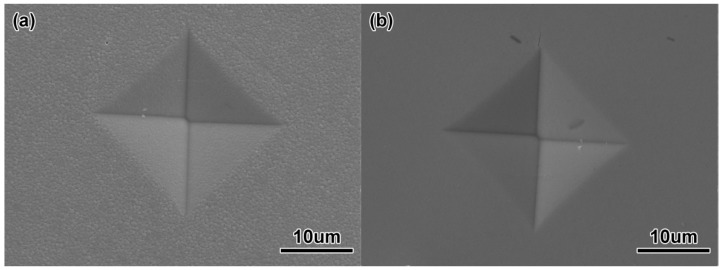
Indentation images of ZrO_2_ samples (**a**) 200 nm, (**b**) 100 nm.

Figure 12 shows the Vickers hardness and bending strength of ZrO_2_ samples with two particle sizes at different sintering temperatures. The Vickers hardness levels of ZrO_2_ samples with 100 nm and 200 nm particle sizes became greater at first as the sintering temperature rose, and the maximum values of 1425.8 HV and 1265.5 HV, respectively, appeared at 1250 °C and 1500 °C. The values fell as the sintering temperature rose. Figure 13 shows the indentation morphology of ZrO_2_ samples with different powder sizes at different sintering temperatures. This change was attributed to the fact that the hardness was mainly affected by the grain size, density, porosity, and micro-cracks after sintering. At lower sintering temperatures, the density was lower and there were some pores in the micro-structure, which made Vickers hardness a little lower. When the sintering temperature increased, the density increased, the porosity decreased, and the grain growth was limited. Therefore, the Vickers hardness increased. When the sintering temperature continued to rise, the grain grew rapidly, and even abnormally so isolated pores were formed at the trigeminal and quadruple grain boundaries, resulting in the decrease of Vickers hardness. Thus, the smaller the grain was, the bigger the Vickers hardness was, and vice versa.

The bending strength of ZrO_2_ samples sintered with different particle sizes increased first and then decreased with the rise in the sintering temperature. With an increase in porosity, the bending strength of ceramic materials decreased exponentially. At lower sintering temperatures, both samples had higher porosity, so the corresponding bending strength values were lower. With an increase in the sintering temperature, the density and the corresponding bending strength increased. For the samples with 100 nm and 200 nm powder sizes, the maximum values were 503.6 MPa and 453.4 MPa at 1250 °C and 1500 °C, respectively. With an increase in grain size, the bending strength decreased remarkably. For the ZrO_2_ samples, an increase in sintering temperature caused rapid and abnormal growth of grains, and the residual pores in grain boundaries and grains which will bring gravitational concentration, so that the bending strength decreased with an increase in the sintering temperature.

**Figure 14:**
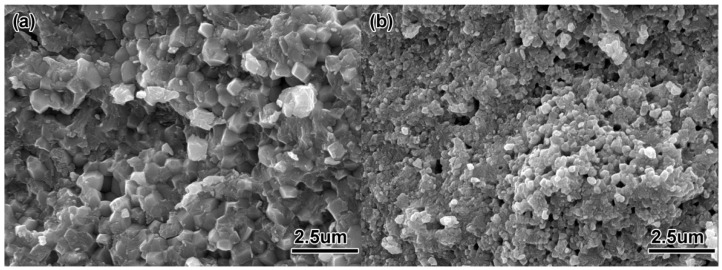
SEM micrographs of the fracture surfaces for sintered ZrO_2_ samples (**a**) fracture surfaces using 200 nm powders, (**b**) fracture surfaces using 100 nm powders.

Figure 14 shows the fracture morphology and crack extension of ZrO_2_ samples with different particle sizes. The fracture morphologies of the two samples were mainly intergranular fracture and mixed fracture mechanisms of intergranular fracture and transgranular fracture, especially when abnormally grown grains were encountered at the front of the crack. Since cracks propagated along the grain boundary for a long time, the stress concentration at the front of the crack was aggravated, which led to transgranular fracture of the large grain and then, sector fracture. Crack propagation along the grain path was more tortuous, coupled with crack tip bridging, and other effects consumed more fracture energy, to obtain better mechanical properties.

### 3.3. Performance Comparison of the Different Powders

Table 4 shows a comparison of the injection molding, debinding and sintering properties of the different powders. In the injection molding stage, because the powder loading of the sample with 100 nm powder was lower than that of the sample with 200 nm powder, there were more voids in the sample with 100 nm powder after injection molding, and the bending strength of the sample with 100 nm powder was lower than that of the sample with 200 nm powder. In the debinding stage, the sample with 100 nm powder exhibited better sintering properties, so it had a higher linear shrinkage and bending strength. In the sintering stage, compared with the sample with 100 nm powder, the sample with 200 nm powder had higher initial powder loading, so it had higher density and a lower linear shrinkage. The results also showed that a nano-scale additive caused the manufactured samples to have increased hardness and strength values. The sintering temperature of samples with 100 nm powder reduced by more than 250 °C under the condition of obtaining nearly complete compact specimens.

### 3.4. Formability of Micro-Parts with the High-Aspect Ratio Micro-Hole Array

These ZrO_2_ nozzles manufactured by micro powder injection moulding in this experiment can be used as the key parts and components of digital ink-jet printing equipment. The main evaluation indexes of the nozzles with the high-aspect ratio micro-hole array in the application were density, mechanical properties, surface roughness of micro-hole, parallelism between micro-holes, and tolerance of micro-holes size. The density and mechanical properties of the test methods and results had been analyzed before. Next, the test methods and results of the other three indicators were introduced.

**Figure 15:**
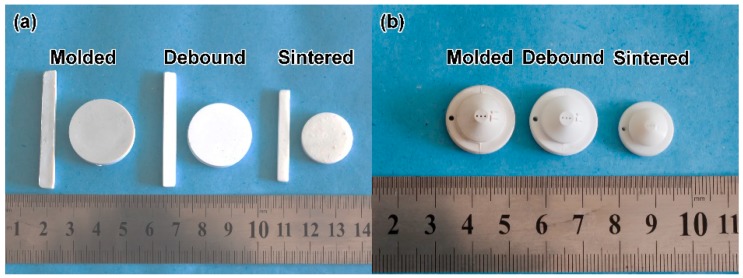
Photographs of molded, debound and sintered (**a**) ZrO_2_ samples, (**b**) ZrO_2_ ceramic nozzles with multi-micro holes.

**Figure 16:**
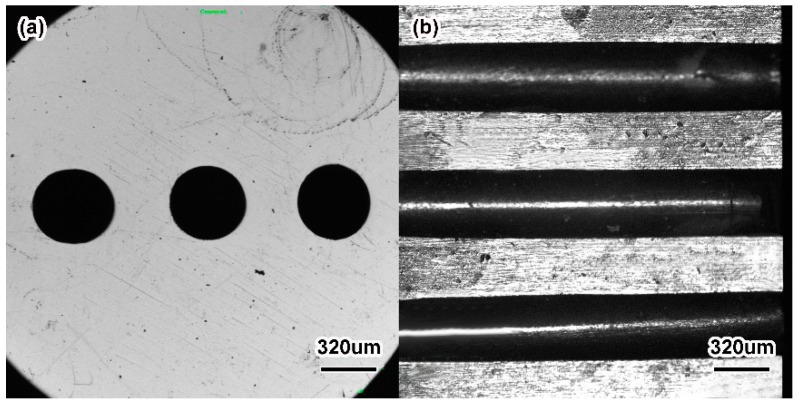
Partial view of sintered micro-holes: (**a**) overhead view; (**b**) cutaway view.

Figure 15 shows the physical map of the ZrO_2_ ceramic mechanical test specimen and ZrO_2_ ceramic nozzles with multi-micro holes after sintering. At all stages, the samples had shape preservation, and there were no macroscopic cracks, warping or other defects. Compared with the billet after injection, the dimensions of the degreased ZrO_2_ ceramic microporous nozzle had less change. The linear shrinkage of the degreased ZrO_2_ ceramic microporous nozzle was between 1% and 2%, but the sintered ZrO_2_ sample had a larger linear shrinkage. Figure 16 shows the local view of the sintered sample. It can be seen that the sintered ceramic micropores had good roundness and high axial parallelism. The size of the micropores after sintering was 450 ± 5 μm, which met the production requirements of the ceramic microporous nozzles. The results told that the micro-powder injection molding technology processed ceramic microporous nozzles with low cost and high efficiency, selected suitable feedstock and powder loading, and designed suitable injection, debinding and sintering processes with higher forming accuracy levels (the dimension error was less than 1.5%).

It was found that the overall size shrinkage of the ZrO_2_ microporous nozzle was basically the same during the sintering. Taking the ZrO_2_ microporous nozzle with 200 nm powder at 1500 °C as an example, the contraction of the nozzle in the axial, radial and circumferential directions was approximately 18.04%, as shown in Table 5. However, it was also found that the linear shrinkages of the micropore and the pore wall between micro-pores were not consistent with the overall shrinkage of the nozzle. The shrinkage of the micropore was smaller than the overall shrinkage of the nozzle, and that of the pore wall was greater than the overall shrinkage of the nozzle. The linear shrinkage of the micropore was smaller than that of the nozzle substrate, mainly because during the filling process of injection molding, the feedstock first entered the whole part of the nozzle, and then filled the pore wall between the micropores, resulting in the sudden decrease of the filling channel. The feedstock had to go through the filling change from a large section to a small section. The mold core subjected the feedstock to a reverse shear stress, which caused ceramic particles in the feedstock into a certain directional arrangement, and led to harder filling owing to the separation of powder and binder and greater feedstock viscosity. The sudden shear stress made a transformation from dense accumulation to loose accumulation when the feedstock was filled through the variable cross-section. On the other hand, an increase in feedstock viscosity weakened the filling pressure of the pore wall. Given what had been mentioned above, the density of micropore wall in the billet after injection was smaller than that at the substrate. In addition, the uneven temperature at the pore wall between the substrate and the micropore might be one of the reasons for this [46]. The density of the micropore wall after injection was lower than that of the whole nozzle. Higher density at the pore wall in the process of sintering densification required greater linear shrinkage. The overall shrinkage of the nozzle was basically the same, while the shrinkage of the pore wall was larger. Therefore, in order to balance the shrinkage of the overall size, the linear shrinkage of the array micropores should be smaller.

**Figure 17:**
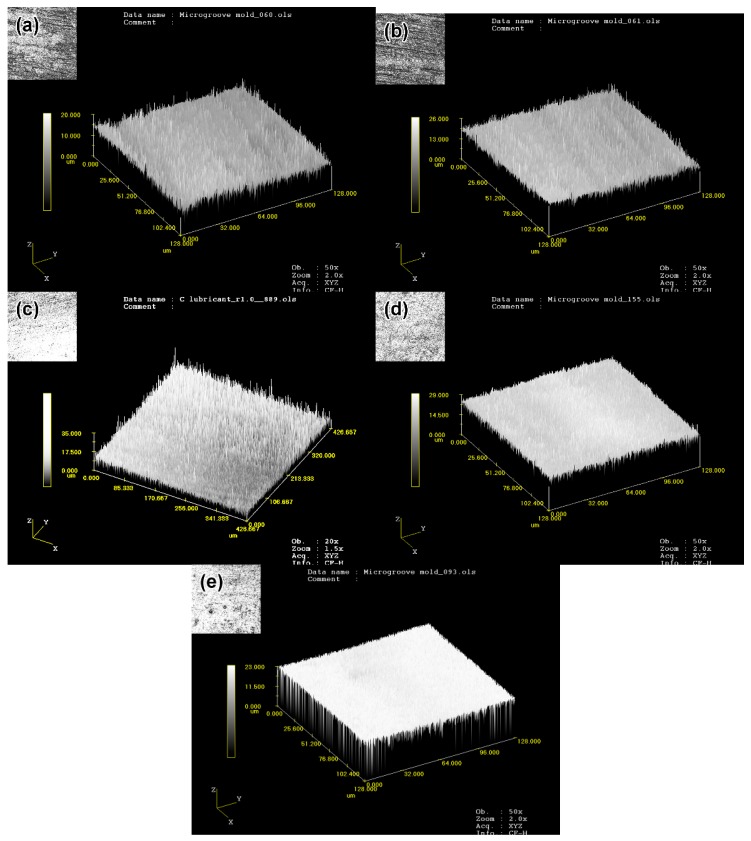
Three-dimensional morphology of ZrO_2_ nozzles with multi-micro-holes at different processing stages (**a**) mold of nozzles, (**b**) molded, (**c**) debound, (**d**) sintered at 1500 °C with 200 nm, (**e**) sintered at 1250 °C with 100 nm.

The surface roughness of ZrO_2_ microporous nozzles at different stages was measured by a LEXTOLS 3000 laser confocal microscope, as shown in Figure 17. The surface roughness of the mold was 0.9 μm, and the injected nozzle was well reproduced with 1.35 μm of the surface roughness. As shown in Figure 17b, the whole surface of the injected nozzle was relatively flat. After debinding, the roughness of the parts increased to 2.45 μm, which was mainly due to the decomposition and volatilization of the binder during debinding and because of the massive holes between the ceramic particles (see Figure 17c). The surface roughness of the parts obtained at different sintering temperatures tended to decrease first and then increase. Mostly, with the rise of the sintering temperature, the density of the parts increased, resulting in better surface roughness. However, the rising temperature later caused grain coarseness and void generation, making the roughness worse. For the sample sintered at 1500 °C with 200 nm powder, the roughness value Ra was 1.92, and for the sample sintered at 1250 °C with 100 nm powder, Ra was 1.32. It was observed that the surface roughness of the sintered part weakened with the decrease of the initial particle size, that is, the surface quality became better. The corresponding surface topography was shown in Figure 17d,e. It was found that ultra-fine ZrO_2_ ceramic powder reduced the sintering temperature and improved surface roughness. However, the decrease in particle size decreased the powder loading and the linear shrinkage of the sintered parts increased, which reduced the precision control of the parts. Moreover, the cost of the raw powder increased with the decreased particle size. Therefore, raw powders with specific particle size should be selected in accordance with the specific requirements of different parts.

## 4. Conclusions

A ceramic microporous nozzle mold with heating and vacuum systems was designed and manufactured. The opening mechanism took the form of a side slider. A thermoplastic system of PW, PP and SA were selected as the binders, and the feedstock prepared by mixing and extrusion granulation processes had fine uniformity and rheological properties. The high-aspect-ratio microporous nozzle was successfully fabricated. After debinding, there were certain sintering characteristics, and the bending strength and linear shrinkage of the parts were 3.78 MPa and 1.45% (for the 200 nm powder), and 11.10 MPa and 1.78% (for the 100 nm powder), respectively. It was found that the smaller the particle size was, the larger the specific surface area was, the higher the specific surface energy was, and the more favorable the sintering densification. The optimum sintering temperature and density of 200 nm the sample were 1500 °C and 99.5%, and those of the 100 nm sample were 1250 °C and 98.36%. The sintering temperature was reduced by 250 °C. The surface roughness of the sintered samples could be effectively improved by ultrafine powder. When the particle size dropped from 200 nm to 100 nm, the roughness value decreased from 1.92 to 1.32. After sintering, the ceramic microporous nozzle had no defects such as cracks, distortion or warping. The micropore was round and the dimension accuracy was less than 1.5%.

## Figures and Tables

**Table 1:** Chemical composition of ZrO_2_ powder (wt.%).

Y_2_O_3_	Al_2_O_3_	SiO_2_	Fe_2_O_3_	MgO	CaO	TiO_2_	Na_2_O
3	≤0.005	≤0.005	≤0.003	≤0.003	≤0.003	≤0.001	≤0.001

**Table 2:** Density deviation of the ZrO_2_ feedstock with different particle sizes.

Samples	Particle Size (nm)	1	2	3	4	5	6	Average Value	Average Deviation
Value	200	−0.1	−0.07	−0.04	−0.11	−0.06	−0.08	−0.077	−2%
	100	−0.25	−0.23	−0.27	−0.26	−0.25	−0.26	−0.25	−7.1%

**Table 3:** Flexural strength and linear shrinkage of the different samples after molding and debinding.

Samples	Particle Size (nm)	Flexural Strength (MPa)	Linear Shrinkage (%)
Molded	200	11.3	-
	100	9.66	-
Debinded	200	6.78	1.45
	100	11.10	1.78

**Table 4:** Comparison of injection, debinding and sintering properties with the different powders.

Samples	Particle Size (nm)	Flexural Strength (MPa)	Linear Shrinkage (%)	Density (%)
Molded	200	11.3	-	-
	100	9.66	-	-
Debinded	200	6.78	1.45	-
	100	11.10	1.78	-
Sintered	200	453.4 (max)	18.04 (max)	99.5 (max)
	100	503.6 (max)	20.52 (max)	98.36 (max)

**Table 5:** Linear shrinkage of ZrO_2_ nozzles with micro holes sintered at 1500 °C (%).

Relative Density (%)	Linear Shrinkage		
	Nozzle	Micro-Hole	Wall of Two Adjacent Holes
99.5	18.04	15.3	21.4
